# Estimating mortality in rare diseases using a population-based registry, 2002 through 2019

**DOI:** 10.1186/s13023-023-02944-7

**Published:** 2023-11-17

**Authors:** Monica Mazzucato, Laura Visonà Dalla Pozza, Cinzia Minichiello, Ema Toto, Andrea Vianello, Paola Facchin

**Affiliations:** 1 Rare Diseases Coordinating Centre, Veneto Region, Padua University Hospital, Padua, Italy; 2 Department of Child and Maternal Health, Padua University Hospital, University of Padova, Padua, Italy

**Keywords:** Rare diseases, Epidemiology, Public health, Mortality, Survival, ORPHAcodes

## Abstract

**Background:**

Rare diseases (RD) are a heterogeneous group of diseases, sharing aspects of complexity. Prognosis is variable, even in individuals with the same disease. Real-world data on RD as a whole are scarce. The aim of this study is to provide data on mortality and survival for a substantial group of RD deriving from a population-based registry, which covers the Veneto region in Italy (4.9 million inhabitants).

**Results:**

During the study period, 3367 deaths occurred, mainly in males (53.9%), elderly patients (63.5%) and patients with diseases having a reported prevalence of 1–9/100000 (65.6%). When standardizing by age, the mortality ratio was higher in RD patients than in the general population, SMR = 1.93 (95% CI 1.84–2.11), with an observed gender difference, 2.01 (95% CI 1.88–2.29) in females and 1.86 (95% CI 1.73–2.10) in males. The lowest survival rates are experienced by patients with rare neurologic diseases, rare skin diseases and rare systemic or rheumatologic diseases, 58%, 68% and 81%, respectively, after a 15-year observation period. It should be noted that only 18% of patients diagnosed with motor neuron diseases were alive after 15 years from diagnosis.

**Conclusions:**

Despite progress in diagnosis, treatment and care in recent years, RD patients globally have higher mortality rates and reduced survival compared to the general population, with specific variations according to gender, age and disease group.

## Introduction

Rare diseases (RD) are widely recognized as a public health priority [1, 2]. Ongoing surveillance and monitoring of these infrequent and heterogeneous conditions are critical to understanding their course as well as their impact both at an individual and population level. Unfortunately, the under-representation of specific RD entities in the coding systems used to produce morbidity and mortality data prevents the visibility of affected patients in health information systems, making this monitoring effort a real challenge [3]. Mortality data are particularly important as they can inform decision makers about the impact of new health problems, such as RD, that are a fairly recent concept in the medical domain [4]. Rare disorders are often reported to have a poor prognosis due to their complexity, the limited knowledge of underlying mechanisms and the scarcity of available etiologic treatments. A number of studies have attempted to estimate the mortality of specific RD [5–13]. More limited information is available on the mortality attributable to selected groups of RD [14–17]. Recently, an Irish study demonstrated that RD cases are over-represented among pediatric in-hospital mortality data [18]. An increased in-hospital mortality has been reported for adult patients as well [19]. A wide range of data sources can be used to derive mortality figures, each having potential limitations. While hospital administrative data only include in-hospital deaths, population-based mortality registries aim to capture all deaths occurring in a defined population, independently of the place of death. Nevertheless, case ascertainment depends on the sensitivity and specificity of the codes used to record diagnoses, which may vary across monitored disease/s, potentially leading to under-reporting [20, 21]. A common limitation is that both these sources are based on the use of the International Classification of Diseases (ICD) [22], a coding system which, regardless of the version used, has a general limited capacity of tracing RD patients. This limitation has been addressed in the recent ICD revision process. ICD-11 will allow a better representation of RD [23], but the effects of this action will only be seen in the long term, when ICD-11 becomes widely used. Disease registries are another important source of mortality data. In these data collections, diagnosis recording is usually accurate and based on defined criteria. Nevertheless, major limitations in using this approach are the long observation period needed to follow-up patients and the fact that the population from which the cases arise is not always well-defined, preventing the calculation of appropriate death rates. In addition, these registries are usually designed to collect data on a specific disease or a group of related diseases, thus hampering the availability of data referred to a significant number of unrelated RD.

The aim of this study is to provide a descriptive analysis of mortality and survival in patients diagnosed with a RD, using data from 2002 to 2019 and deriving from a population-based registry established in the Veneto region, Italy.

## Materials and methods

The study population included all the residents of the Veneto, a region located in the northeast of Italy with a population of 4,905,854 [24]. The Veneto region has an increasingly aging population due to the low birth rate and a life expectancy of nearly 80 and 85 years in males and females, respectively. The Italian National Health Service is organized at central, regional and local level and provides universal coverage to all residents. In the Veneto region, 9 local health units (LHUs) provide primary, outpatient and hospital care. Specialist care is also provided by two University Hospitals, four Institutes of research and care (IRCCS) and accredited private providers. The data sources used for this study are the Veneto region RD registry and the Veneto region administrative health database (AHD). The Veneto region AHD consists of an archive of potential healthcare beneficiaries and includes the demographic information and residential history of all the subjects living in the region. Information on the vital status of each resident is also reported. In addition, for each resident, the AHD collects information on exemption from co-payment related to specific conditions, as established by the law, including RD [25]. The AHD is linked via a secure connection to the regional RD registry, allowing the communication of the RD exemption status and the exchange between the two data sources of real-time information on demographic data, such as the date of birth, place of residence and vital status of the enrolled patients. The RD Registry is the primary source of data for RD exemption, whilst the AHD is the primary source of data for the vital status of the patient. A common individual code is used to guarantee the interoperability between the two data sources according to Health Information Exchange standards. The operation of the Veneto region RD registry was described in a previous article [26]. In 2002, a web-based registry was established to collect data on patients diagnosed with a RD from the Italian list of RD. The national RD list defines which patients affected by a RD are entitled to particular benefits, namely exemption from healthcare costs related to the RD diagnosed. The registry connects centers of expertise, local health units, other regional hospitals, rehabilitation facilities and pharmaceuticals services. Currently, in the Veneto Region 1224 health-care professionals working in RD centers of expertise, local health units, and hospital pharmacies access the system. Patients can be enrolled in the RD registry only when clinicians working in centers of expertise, which are officially identified by the regional Health Authority and thus part of the national RD care network, make a RD diagnosis. In the Veneto region, centers of expertise, identified by groups of RD, have undergone four revision processes to date, based on activity data and the analysis of other independent data sources. It should be noted that seven regional hospitals are full members of 1–22 European Reference Networks (ERNs) [27]. For this study, we have considered only RD included both in the Italian official list [25], last updated in 2017, and in the Orphanet nomenclature of RD version 2022 [28].

We have excluded rare cancers and rare infectious diseases, as they are particularly under-represented in the Italian RD list. When considering prevalence ranges for the RD included in the study, we derived data from Orphadata and followed the same methodology described in a previously published article [29]. The prevalence categories considered (< 1/1,000,000; 1–9/1,000,000; 1–9/100,000; and 1–5/10,000) correspond with the ones listed in the Orphanet nomenclature. In the RD registry, each disease entity is assigned the following codes: ICD9-CM, ICD-10, OMIM and ORPHAcodes. ICD-10, ORPHAcodes and OMIM codes are derived from the Orphanet nomenclature pack (version 2022). ICD9-CM codes are still used in Italy for morbidity recording. ICD9-CM codes, which are not available from Orphanet, have been assigned to monitored RD entities by the medical team of the registry considering the same rules adopted by Orphanet for ICD-10 cross-referencing [30]. The mortality data from the RD population were compared with mortality data from the general population of the Veneto region in the same period. Each patient included in the RD regional registry was followed from date of first diagnosis of a RD until 31 December 2019, date of death or date of emigration outside the region. Information on date of death and emigration was obtained from the Regional AHD.

Crude annual mortality rates were calculated by gender and the following age categories (age 0–11 months, 1–4, 5–9, 10–14, 15–19, 20–29, 30–39, 40–49, 50–59, 60–69, 70–79, 80–89 and ≥ 90 years). For the year 2019, the crude annual mortality rate from all causes in RD patients was calculated by dividing the number of RD patients who had deceased in the year by the total population at risk (i.e. the total number of RD patients) per 100,000 individuals.

Furthermore, data were categorized by Orphanet classifications for aggregation and analyses using the information on the preferential parent derived from the Orphanet nomenclature version 2022. In the case of patients diagnosed with two different RD during the study period, if the corresponding preferential parents were different, they were considered separately in the Orphanet classifications analysis. The observed number of deaths was compared with the expected number of deaths derived from gender-, age- and period-specific mortality rates for the general population of the Veneto region, using data published by ISTAT [24]. Gender-specific standardized mortality ratios (SMR) were computed (with 95% CIs), considering the Veneto region population data as the reference. Data are presented grouping RD according to the Orphanet classification. RD patients’ survival was assessed using the Kaplan–Meier survival curve analysis (with 95% CIs) by sex and by Orphanet classification, providing additional data referred to specific disease subgroups (chromosomal anomalies, lysosomal diseases, mitochondrial diseases and motor neuron diseases). Patients diagnosed with a RD and resident in the study area were followed-up within the RD registry. Follow-up started at the date of diagnosis until death, migration outside the study area or end of the study period (December 31st, 2019). All the analyses were carried out on records undergoing the same anonymization process allowing the linkage between the RD Registry and the AHD, without any possibility of back-retrieving the identity of patients.

### Statistical analysis

Statistical analysis was performed with the SAS package, rel. 9.4 (SAS Institute Inc., Cary, NC, USA).

### Ethical issues

The study was conducted on data routinely collected by the health services and anonymized preventing identification of the individual concerned. Data analysis was carried out on anonymized aggregated data.

## Results

**Fig. 1:**
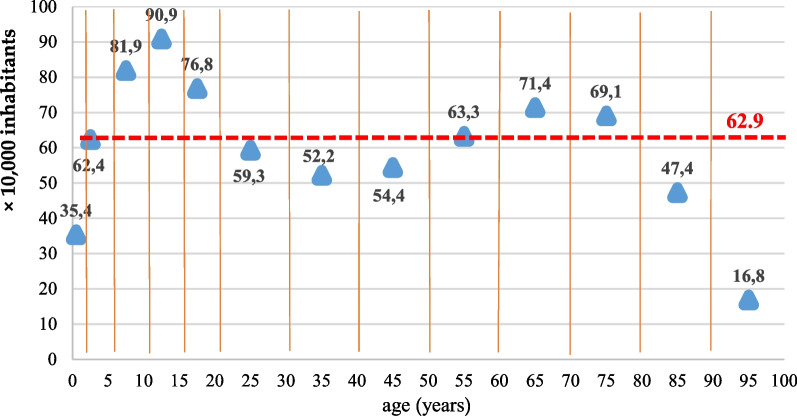
Prevalence per 10,000 by age group (for the 0–11 months age group rates per 1000 live births are reported). Data as of December 31, 2019

In the study area there were 30,879 patients alive as of 31 December 2019; 53.8% were female and 19.9% were pediatric patients (< 18 years). A subset of patients (n = 269) was reported to have two different rare diseases. The overall prevalence of RD in the monitored population was 62.93 per 10,000 inhabitants (95% CI 62.23–63.63), with value fluctuations according to the age class considered (Fig. 1). Two prevalence peaks can be observed, one in the 10–14 years age group (90.87 per 10,000 inhabitants) and one in the 60–69 years age group (71.41 per 10,000 inhabitants).

A higher prevalence was observed in the female patients than in the male patients, 66.31 per 10,000 (95% CI 65.30–67.31) and 59.41 per 10,000 (95% CI 58.44–60.38), respectively (Table 1). Mortality rate in the entire Italian population in 2019 was 9.97 per 1000 residents, 9.61 in men and 10.30 in women. The overall crude mortality rate in RD patients was 8.87 per 100,000 inhabitants (95% CI 8.03–9.70), which was higher in women than in men, 9.06/100,000 (95% CI 7.88–10.24) and 8.66/100,000 (95% CI 7.48–9.84). The mortality rate shows increasing values in the older age groups, with a pediatric peak in the 1–4 years age group (4.56 per 100,000; 95% CI 1.18–7.94) (Table 1). The mortality ratio standardized by age is 1.93 (95% CI 1.84–2.11), higher for females 2.01 (95% CI 1.88–2.29) than for males 1.86 (95% CI 1.73–2.10).

**Table 1:** Prevalence of RD cases as of December 31, 2019 and crude mortality rates (year 2019) by gender and age group

	Prevalence per 10,000—as of December 31, 2019		Mortality rate per 100,000—Year 2019	
		95% CI		95% CI
Total	62.93	62.23–63.63	8.87	8.03–9.70
*Gender*				
Male	59.41	58.44–60.38	8.66	7.48–9.84
Female	66.31	65.30–67.31	9.06	7.88–10.24
*Age*				
0–12 months	35.37	29.11–41.64	0.15*	0.02*–0.28*
1–4 years	62.38	58.44–66.32	4.56	1.18–7.94
5–9 years	81.94	78.18–85.70	1.36	0.00–2.89
10–14 years	90.87	87.05–94.70	3.38	1.04–5.72
15–19 years	76.82	73.28–80.36	0.43	0.00–1.26
20–29 years	59.32	57.16–61.49	0.62	0.00–1.32
30–39 years	52.24	50.32–54.16	1.11	0.22–1.99
40–49 years	54.43	52.78–56.09	2.90	1.69–4.12
50–59 years	63.31	61.57–65.05	5.02	3.47–6.58
60–69 years	71.41	69.28–73.54	14.49	11.45–17.54
70–79 years	69.08	66.77–71.39	28.34	23.65–33.04
80–89 years	47.36	44.85–49.86	34.32	27.56–41.08
90 + years	16.84	13.69–20.00	21.64	10.30–32.97

*Rate per 1000 live births

**Fig. 2:**
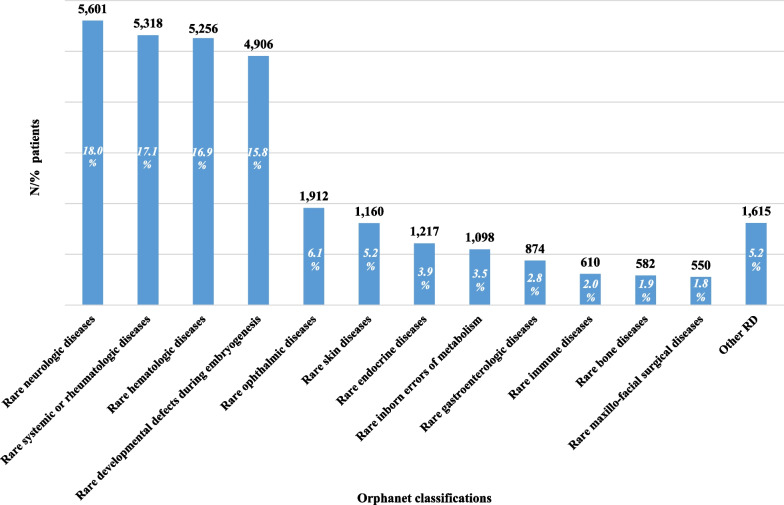
Frequency and percentage distribution of RD patients alive as of December 31, 2019 by Orphanet classification in decreasing order

Patients alive as of December 31, 2019 were mainly diagnosed with rare neurologic diseases (18%), rare systemic or rheumatologic diseases (17.1%), rare hematologic diseases (16.9%) and rare developmental defects during embryogenesis (15.8%) (Fig. 2).

**Fig. 3:**
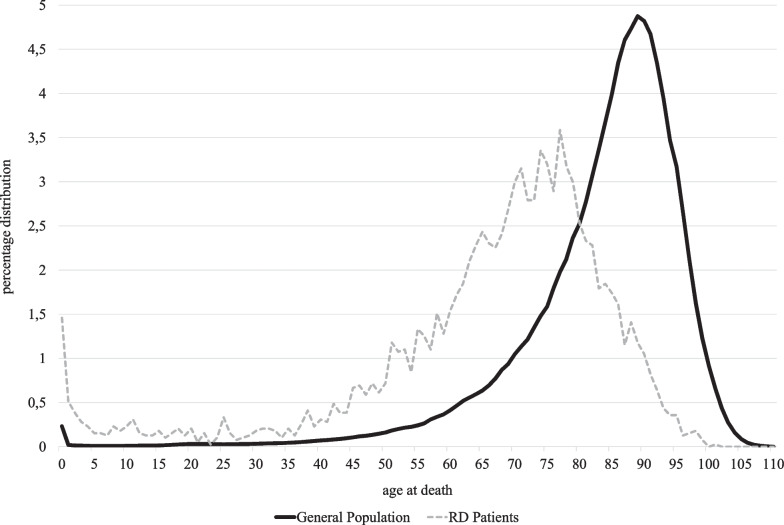
Distribution of age at death in RD patients and the general population (the Veneto region RD registry/National Institute of Health, 2002–2019)

A total of 3367 patients died during the study period. The deaths occurred mainly in male patients (n = 1816; 53.9%) and in patients older than 65 years old (n = 2140; 63.5%). In a minority of cases (n = 190; 5.6%), pediatric patients died. It should be noted that the distribution of the RD patients’ age at death differed from that of the general population. RD patients are likely to die earlier than the general population, mainly in the first years of life and the older age groups (Fig. 3).

The data have been analyzed by disease prevalence class, when available, as derived from Orphanet according to the methods described in Wakap et al. 2020.

**Fig. 4:**
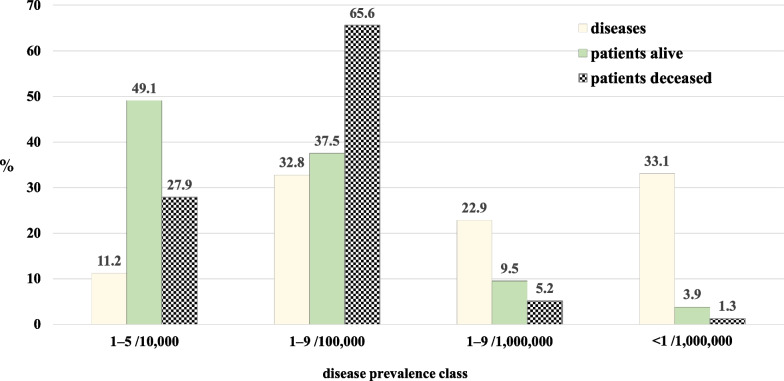
Percentage distribution of RD, RD patients alive and RD patients deceased as of December 31, 2019 by disease prevalence class

Nearly half of the patients alive as of December 31, 2019 had a rare disease with a prevalence of 1–5/10,000 (49.1%), 37.5% had a disease with a reported prevalence of 1–9/100,000 and 13.4% had a very rare disease (prevalence ≤ 9/1,000,000 inhabitants). This distribution differs if we consider patients who died during the study period. The majority (65.6%) was affected by a disease with a reported prevalence of 1–9/100,000, 27.9% had a disease with a prevalence close to the rarity threshold (1–5/10,000). A limited proportion (6.5%) had a very rare disease (prevalence ≤ 9/1,000,000 inhabitants) (Fig. 4).

Considering all the patients deceased during the study period and their corresponding RD diagnoses distribution by Orphanet classification, more than half had a rare neurologic disease (n = 1736; 51.6%). Other represented groups of diseases were rare systemic or rheumatologic diseases (n = 441; 13.1%), rare skin diseases (n = 386; 11.5%) and rare hematologic diseases (n = 255; 7.6%) (Table 2).

**Table 2:** Distribution of diagnoses of patients alive as of December 31, 2019, prevalence and mortality rates by Orphanet classification

Orphanet classification	% patients alive as of December 31, 2019	Prevalence as of December 31, 2019 per 10,000	Mortality rate year 2019 per 100,000
Rare neurologic diseases	18.0	11.41	4.11
Rare systemic or rheumatologic diseases	17.1	10.84	1.74
Rare hematologic diseases	16.9	10.71	0.48
Rare developmental defects during embryogenesis	15.8	10.00	0.26
Rare ophthalmic disorders	6.1	3.90	0.22
Rare skin diseases	5.2	3.28	0.79
Rare endocrine diseases	3.9	2.48	0.06
Rare inborn errors of metabolism	3.5	2.24	0.24
Rare gastroenterological diseases	2.8	1.78	0.16
Rare immune diseases	2.0	1.24	0.18
Rare bone diseases	1.9	1.19	0.02
Rare maxillo-facial surgical diseases	1.8	1.12	0.00
Rare respiratory diseases	1.3	0.85	0.49
Rare hepatic diseases	1.2	0.78	0.08
Rare renal diseases	1.1	0.70	0.00
Rare urogenital diseases	0.8	0.50	0.04
Rare abdominal surgical diseases	0.3	0.18	0.00
Rare circulatory system diseases	0.2	0.15	0.00
Rare otorhinolaryngologic diseases	0.2	0.13	0.00
Rare infertility	0.0	0.02	0.00
Total	100	62.93	8.87

**Fig. 5:**
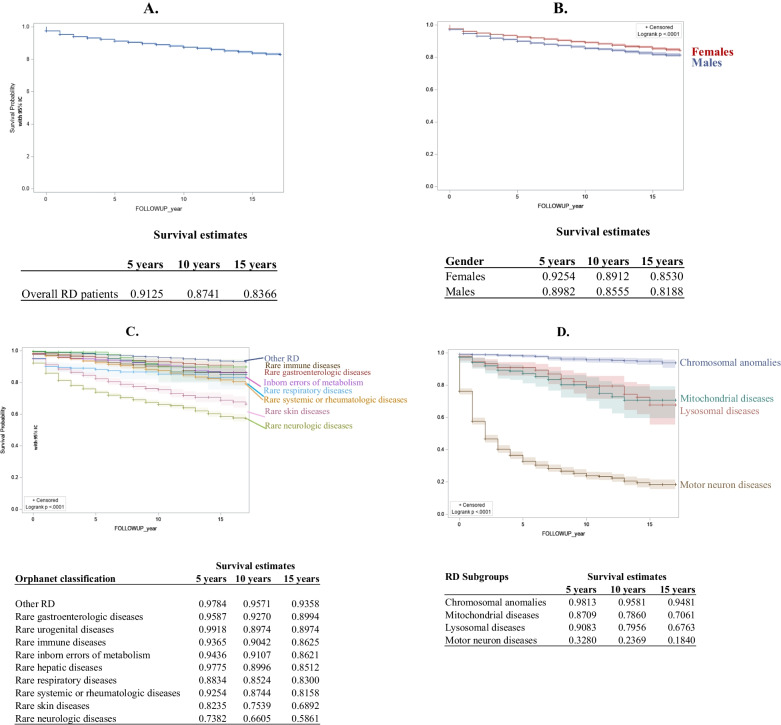
Overall RD patient survival (**a**), by sex (**b**), by Orphanet classification (**c**), by groups of diseases (chromosomal anomalies, lysosomal storage diseases, motor neuron diseases, mitochondrial diseases) (**d**)

Survival at 5, 10 and 15 years from the date of enrollment in the registry for the entire RD population studied, by sex and Orphanet classification, is reported in Fig. 5. Overall survival was 91% at 5 years, 87% at 10 years and 83% at 15 years. Our findings show that survival was lower in the male than the female patients, 81% and 85% at 15 years, respectively. Patients with a diagnosis of a rare neurologic disease, rare skin disease and rare systemic or rheumatologic disease had the lowest long-term survival rate, 58%, 68% and 81%, respectively, after 15 years of observation. When analyzing the specific contribution of disease subgroups to these findings, motor neuron diseases were found to be the diseases with the lowest survival. Only 18% of the patients with these conditions, namely adults diagnosed with amyotrophic lateral sclerosis (ALS) and children with spinal muscular atrophies (SMA), survived at the end of a 15-year observation period. The overall survival rate of patients diagnosed with inborn errors of metabolism was 86% at 15 years. Nevertheless, if we consider disease subgroups within this broader category, such as lysosomal storage diseases and mitochondrial diseases, a lower proportion of patients survived at 15 years, 67% and 70%, respectively.

## Discussion

In this study, we described mortality figures and survival referring to a substantial number of patients diagnosed with a rare disease, and who were enrolled in a population-based registry. Mortality data and observed trends over time are particularly informative as they can document outcomes over the patients’ lifetime and/or areas of unmet needs requiring investments in research and care. Our study showed that RD patients experience higher crude mortality rates than the general population, especially in the older age groups. This finding is in line with previous figures provided by our registry referring to a 10-year observation period [26] and with results from a study carried out in another Italian region, where a similar RD registry has been established [31]. Mortality rates progressively increasing with age can be explained by the accumulation of damage involving many vital systems and organs and by the occurrence of elderly-onset comorbidities, both in the RD and general population. Unfortunately, no information was available in our study on the presence or absence of comorbidities, potentially affecting the observed trends. The observed pediatric mortality peak in the age group of 1–4 years old (4.56 per 100,000) is in relative agreement with evidence from an Irish hospital-based study reporting a significant contribution of RD to pediatric mortality [18]. Interestingly, when standardizing for age, female patients were found to have an increased risk of death than their male counterparts, compared to the general population, 2.01 versus 1.86. This gender difference certainly deserves further study to better understand the intertwining of purely biological factors and factors related to delayed diagnosis and access to treatment and care, as reported in women affected by other conditions.

Our study showed a diverse contribution of different rare disease groups to mortality figures in the monitored population. Patients with diseases belonging to the following Orphanet classifications emerged as the ones affected by higher mortality rates: rare neurologic diseases, systemic or rheumatologic diseases and rare skin diseases.

These findings are not directly comparable with the ones based on data derived from our Registry from 2002 to 2012 for three main reasons. First, the list of RD that undergo mandatory monitoring in Italy was updated in 2017, introducing new RD entities, such as respiratory diseases (i.e. sarcoidosis, idiopathic pulmonary fibrosis, idiopathic pulmonary hypertension) and rheumatologic diseases such as systemic sclerosis, impacting overall and group-related mortality figures. Second, since 2012 we have witnessed impressive changes in the preventive, diagnostic and care field, such as the use of innovative genetic tests, the implementation of neonatal screening policies, and the availability of innovative treatments, although this is still only for a limited proportion of RD. The effects of all these changes may have impacted the survival of the monitored population, especially in selected disease groups. Third, in our previous study and the study exploring RD patient survival in the Tuscany region, disease categorization was based on ICD groups.

As RD are under-represented in ICD, they are usually under-reported in mortality studies. In the forthcoming ICD-11 this problem will be mitigated, allowing in the future to properly estimate RD contribution to mortality in the general population. In the meantime, the use of ORPHAcodes, a specific RD coding resource, helps provide a snapshot of the contribution of diverse disease groups to RD epidemiologic figures.

Thanks to the use of ORPHAcodes, we were able to highlight the impact on patient survival of specific subgroups of diseases within broader categories, as is the case of lysosomal storage diseases. Although in some cases neonatal screening policies include these diseases, and despite the availability of innovative treatments, it is important to report that affected patients still experience reduced survival.

Another group of patients presenting highly reduced survival rates are those diagnosed with rare neurologic diseases. This is mainly attributable to adult and elderly patients with ALS and to pediatric patients diagnosed with SMA. Several studies have investigated ALS survival in selected populations, using data either from disease registries or from population-based registries [32].

Our study findings are in accordance with previous studies; however, we also identify ALS contribution to overall mortality figures referring to a wider RD population.

Along with ALS patients, SMA patients experience a severe disease course, especially SMA type I patients, for whom a median life expectancy of less than 2 years without respiratory support has been reported [33]. This disease trajectory is confirmed by our study.

According to our study findings, overall, only 18% of patients diagnosed with motor neuron diseases were alive after a 15-years observation period.

Recently, SMA patients have benefitted from the availability of new disease-modifying treatments, in particular SMA type I children. Along with these new therapies, neonatal screening for this condition is already performed in some countries/regions, and is under discussion in others [34]. Some evidence of survival benefit following the introduction of these therapies, besides clinical trial data, is available [35]. Nevertheless, more data are needed from real-world population-based studies with long-time observation periods so that a comparison can be made between cases before and after (1) the introduction of neonatal screening and (2) access to these therapeutic options.

The same applies to rare respiratory diseases, a group of diseases which generally affects adult and elderly patients and is among the diseases presenting reduced patient survival in the present study. To what extent new available therapies and improved access to lung transplantation has an impact on patients’ long-term survival is an important research question requiring further study.

Although rare skin diseases were diagnosed in a minority (5.2%) of all the patients enrolled in the Registry during the study period, very poor survival rates can be observed in this subset of patients. This is in line with previous studies investigating survival, although in distinct rare skin diseases [36, 37]. What this study adds is their overall contribution to mortality figures in the population under study.

## Strengths of the study

The major strengths of this study are its population-based approach and the long study period considered, allowing the follow-up of a relatively large group of patients diagnosed with different RD. We analyzed data from a population of 4.9 million inhabitants, roughly representing 10% of the Italian population, for an extended period of time (2002–2019). Because we studied mortality using a population-based approach, our surveillance should be comprehensive, mitigating potential biases introduced when estimating mortality from hospital-based populations. The enrollment in the RD registry is the prerequisite for exemption from any healthcare costs related to the RD diagnosed. It is therefore conceivable that the RD registry has a good coverage. Nevertheless, there may have been a certain level of incompleteness in case ascertainment in the first years of operation of the registry, and following the update of the list of RD monitored in 2017, as time was needed to enroll new and previously-diagnosed patients in the registry. Although the monitored diseases do not include all the disease entities in the Orphanet nomenclature, they represent a substantial group of rare conditions, belonging to almost all the medical domains. It should be noted that some disease areas are affected by under-representation in the Italian official RD list (i.e. rare tumors and rare infectious diseases) and thus they have not been considered in the present study. Given the population-based approach of the study and the data sources used, we were able to record all deaths in the study period, independently of the place in which they occurred.

An additional strength is that clinicians working in tertiary referral centers, which are labelled as of expertise for specific groups of RD following an official selection process, are responsible for the enrollment of RD patients in the registry. Thus, the RD diagnosis is expected to be accurate. Moreover, as diagnoses are recorded based on the use of ORPHAcodes, a specific RD coding resource, the level of diagnostic detail is higher than that available from studies based on data from an administrative database.

In addition, unlike other studies in which ORPHAcodes have been attributed to RD patients starting from ICD codes and reviewing health records, in the present study they are assigned at the point of diagnosis/care by RD experts, reducing potential codification biases.

## Limits

The limitations of this study mainly depend on the characteristics of the data used. The limits of the Veneto region RD registry as a source of data for epidemiologic studies have been already described [26].

Other limitations are intrinsically related to the study design. As we could not review the death certificates of RD patients enrolled in the registry who died during the study period, we were not able to investigate RD as a specific cause of death or consider the role of other co-morbidities in the causal chain of death. However, as RD are under-represented in the ICD-10, which is used in Italy to compile death certificates, we can assume that, even if this data source were accessible, the under-reporting of RD may lead to an underestimation of their contribution to the observed mortality figures. This has already been reported in some studies investigating RD mortality based on death certificates [20, 21].

Another limit refers to the possible underestimation of the contribution to mortality figures of specific groups of diseases, namely congenital anomalies, and other severe diseases having a perinatal onset. Patients with such conditions may have not been enrolled in the registry due to early mortality.

Finally, the study was carried out before the start of the SARS-CoV-2 outbreak. As some studies have reported increased mortality in RD patients during the pandemic, further research is needed to evaluate the impact of the global emergency on this vulnerable population [38–40].

## Conclusions

RD share many characteristics, but are rarely studied as a category. RD patients experience higher mortality rates compared to the general population. Further research is needed to understand the mechanisms determining mortality in RD patients and especially in subgroups presenting higher death rates. The use of a RD specific coding resource such as ORPHAcodes can better identify these diseases as a distinct, although composite category allowing their associated burden to be estimated. This could serve as the basis to develop further targeted healthcare policies to tackle the needs of the most vulnerable groups of patients among the RD population.

## Data Availability

The data supporting this study's findings are available from the corresponding authors [MM, PF], upon reasoned request and with the permission of the Veneto region.
