# Correction: Estimating mortality in rare diseases using a population-based registry, 2002 through 2019

**DOI:** 10.1186/s13023-024-03051-x

**Published:** 2024-02-06

**Authors:** Monica Mazzucato, Laura Visonà Dalla Pozza, Cinzia Minichiello, Ema Toto, Andrea Vianello, Paola Facchin

**Affiliations:** 1 Rare Diseases Coordinating Centre, Veneto Region, Padua University Hospital, Padua, Italy; 2 Department of Child and Maternal Health, Padua University Hospital, University of Padova, Padua, Italy

**Correction: Orphanet Journal of Rare Diseases (2023) 18:362** 10.1186/s13023-023-02944-7

Following publication of the original article [1], we have been notified that the article Figure 5 is missing its parts C and D. Figure 5 was published as per below:

**Fig. 5:**
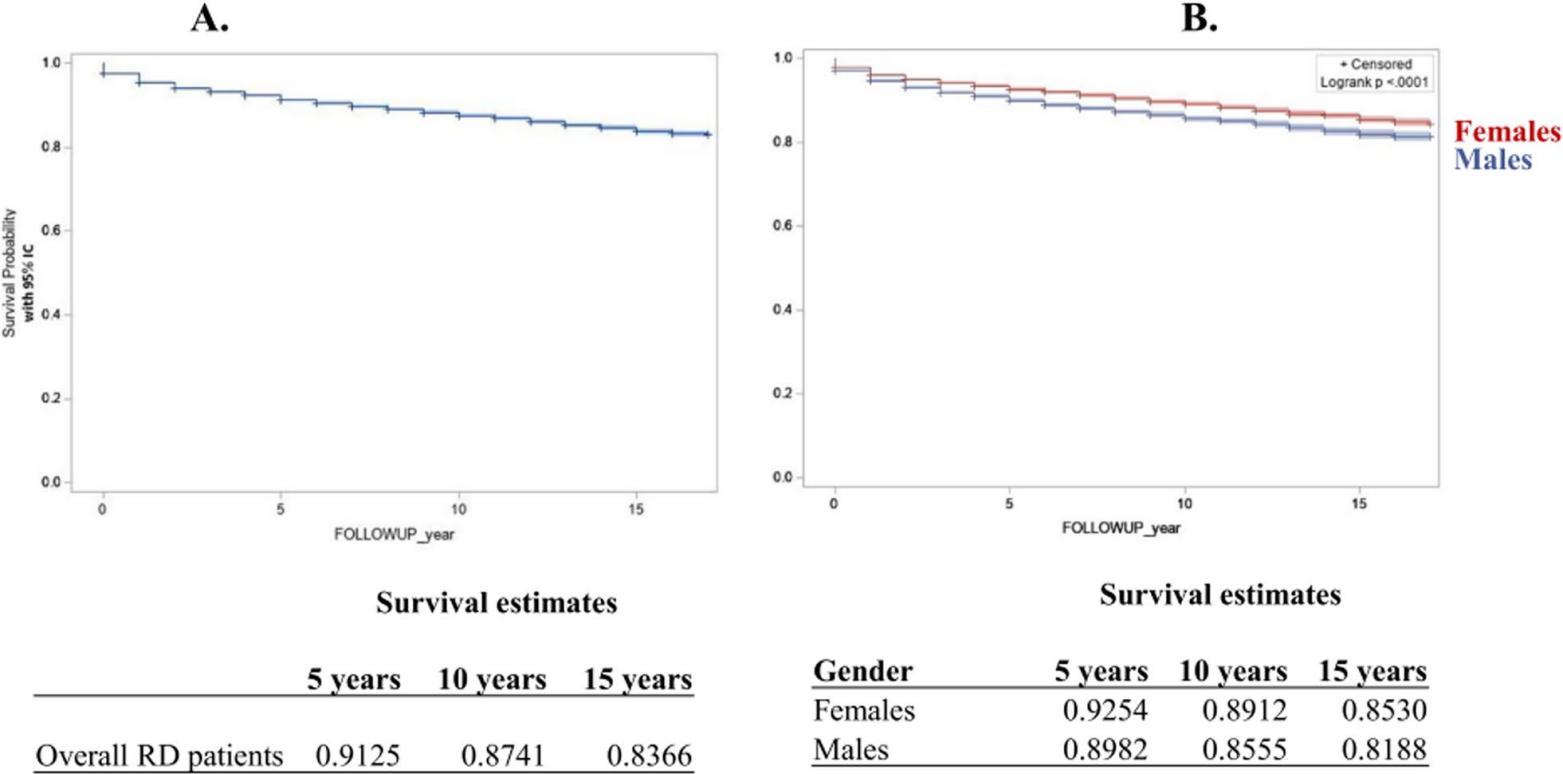
Overall RD patient survival (**a**), by sex (**b**), by Orphanet classification (**c**), by groups of diseases (chromosomal anomalies, lysosomal storage diseases, motor neuron diseases, mitochondrial diseases) (**d**)

It should be as follows:

**Fig. 5:**
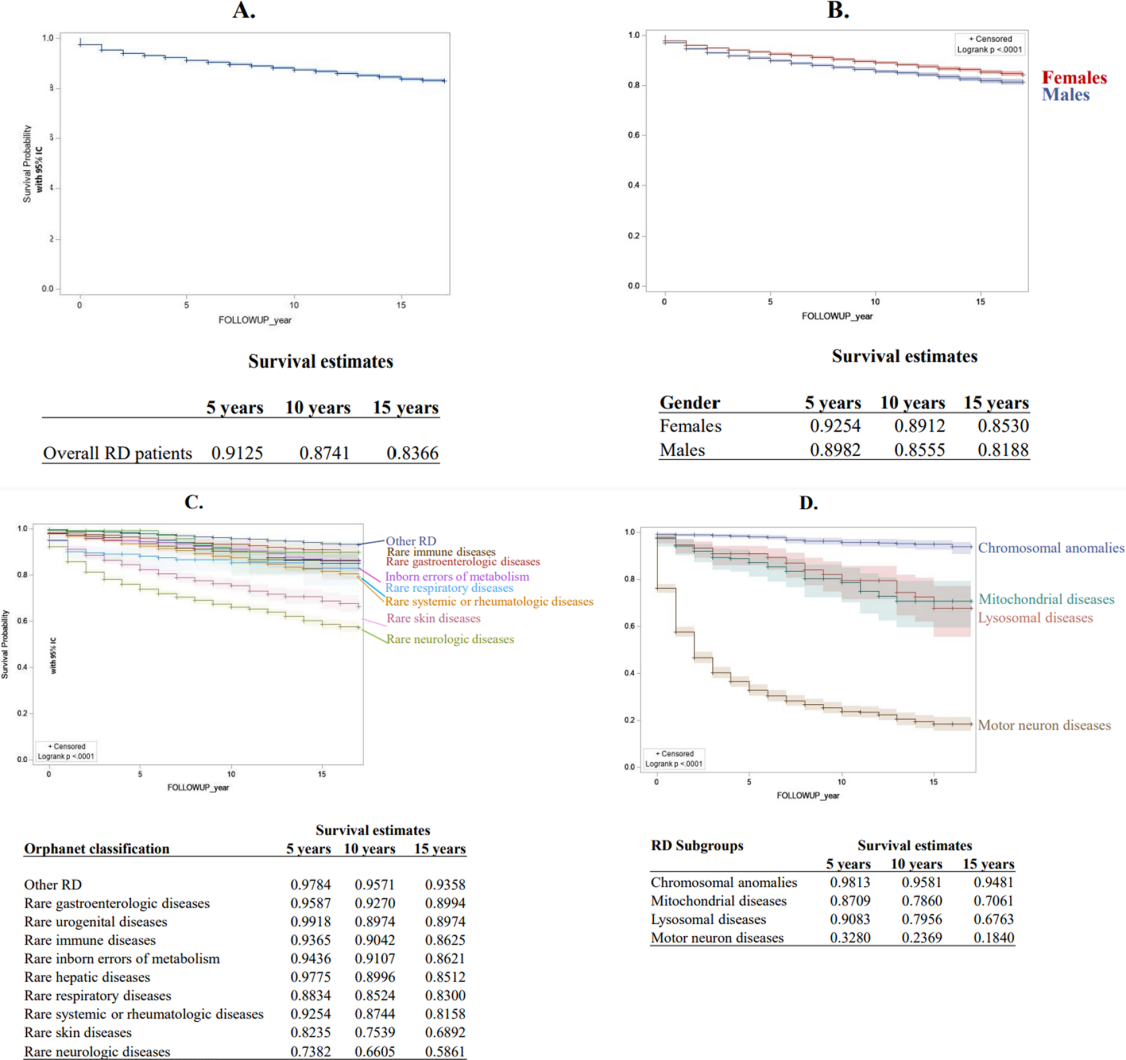
Overall RD patient survival (**a**), by sex (**b**), by Orphanet classification (**c**), by groups of diseases (chromosomal anomalies, lysosomal storage diseases, motor neuron diseases, mitochondrial diseases) (**d**)

The original article was updated.
